# The prediction of suicide in severe mental illness: development and validation of a clinical prediction rule (OxMIS)

**DOI:** 10.1038/s41398-019-0428-3

**Published:** 2019-02-25

**Authors:** Seena Fazel, Achim Wolf, Henrik Larsson, Susan Mallett, Thomas R. Fanshawe

**Affiliations:** 1 Department of Psychiatry, Warneford Hospital, University of Oxford, Oxford, UK; 2 Department of Medical Epidemiology and Biostatistics, Karolinska Institutet, Stockholm, Sweden; 3 School of Medical Sciences, Örebro University, Örebro, Sweden; 4 School of Population and Health Sciences, University of Birmingham, Birmingham, UK; 5 Nuffield Department of Primary Care Health Sciences, University of Oxford, Oxford, UK

## Abstract

Assessment of suicide risk in individuals with severe mental illness is currently inconsistent, and based on clinical decision-making with or without tools developed for other purposes. We aimed to develop and validate a predictive model for suicide using data from linked population-based registers in individuals with severe mental illness. A national cohort of 75,158 Swedish individuals aged 15–65 with a diagnosis of severe mental illness (schizophrenia-spectrum disorders, and bipolar disorder) with 574,018 clinical patient episodes between 2001 and 2008, split into development (58,771 patients, 494 suicides) and external validation (16,387 patients, 139 suicides) samples. A multivariable derivation model was developed to determine the strength of pre-specified routinely collected socio-demographic and clinical risk factors, and then tested in external validation. We measured discrimination and calibration for prediction of suicide at 1 year using specified risk cut-offs. A 17-item clinical risk prediction model for suicide was developed and showed moderately good measures of discrimination (c-index 0.71) and calibration. For risk of suicide at 1 year, using a pre-specified 1% cut-off, sensitivity was 55% (95% confidence interval [CI] 47–63%) and specificity was 75% (95% CI 74–75%). Positive and negative predictive values were 2% and 99%, respectively. The model was used to generate a simple freely available web-based probability-based risk calculator (Oxford Mental Illness and Suicide tool or OxMIS) without categorical cut-offs. A scalable prediction score for suicide in individuals with severe mental illness is feasible. If validated in other samples and linked to effective interventions, using a probability score may assist clinical decision-making.

## Introduction

Suicide risk assessment is currently a central component of clinical care in psychiatry^1–3^, and conducted in individuals who present to primary and secondary health services with psychiatric symptoms. Many different structured tools are used to assist in clinical decision-making, most of which have not been validated in external data^4^, and some of which have poor accuracy^5^, potentially increasing workload of clinical teams by identifying false positives. As a consequence, some experts have suggested that such tools should not be used, and clinical judgement be solely used for suicide risk assessment. Further, it has been argued that identifying high-risk groups might detract from providing evidence-based clinical care to all patients, which could be a more effective strategy to reduce suicide rates^6^.

However, it remains uncertain whether the alternative—unstructured clinical approaches without the use of risk-assessment tools—lead to improved prediction and outcomes. It has been argued that such tools also provide a useful baseline assessment, a checklist to identify risk factors, and can be linked to interventions for higher risk individuals^7,8^. In the US, tools are recommended by the National Strategy for Suicide Prevention^9^, and the European Psychiatric Association has suggested that they act as adjuncts for clinical assessment^10^, while in England^11^, Australia and New Zealand^12^, guidelines argue for a needs-based assessment over risk assessment for individuals who present with self-harm. A recent expert clinical review concludes that new structured approaches need to be developed and researched, especially for completed suicide as an outcome^8^. One important population with high suicide risks for which there are no specific tools are individuals with a severe mental (or psychotic) illness, namely schizophrenia-spectrum disorders and bipolar disorder^13^. The risk of suicide in schizophrenia is around 20 times higher than in the general population^14^, contributing to the estimated average 15 years of potential life lost^15^, with a range of modifiable risk factors^16^. In individuals with bipolar disorder, a systematic review reported a pooled standardised suicide mortality ratio of 17 ^17^. In this study, we aim to develop and externally validate a risk-assessment tool for suicide in individuals with schizophrenia-spectrum or bipolar disorder.

## Methods

### Study sample

We conducted a cohort study of individuals aged 15–65 with a diagnosis of severe mental illness through linkage of population-based registers in Sweden divided into derivation and external validation datasets. We included schizophrenia-spectrum disorders (ICD-8: 295, 297–299; ICD-9: 295, 297–299 excl. 299A; ICD-10: F20–F29) and bipolar disorders (296 excl. 296.2; 296 excl. 296D; F30–F31) diagnoses in the National Patient Register. We identified a cohort of 75,158 individuals with 574,018 recorded patient episodes between 1 January 2001 and 31 December 2008. The final study cohort consisted of a single inpatient or outpatient visit for each patient, selected at random with equal probability, to create a model that can be used for any patient episode. Repeat visits were excluded as these would complicate interpretation and model fitting, and we envisaged the tool being used on a single occasion for each individual rather than repeatedly over time. Each individual was followed from the day of discharge until death, emigration or end of follow-up (12 months post-discharge).

### Measurement of risk factors

Individuals within the cohort were linked to national registers to obtain information on risk factors, with unique personal identification numbers enabling accurate linkage^18^. From the Total Population Register^19^ and Longitudinal Integration Database for Health Insurance and Social Studies, we obtained socio-demographic factors. We identified psychiatric diagnoses from the National Patient Register^20^, and obtained data on dispensed medication from the Swedish Prescribed Drug Register^21^. We further identified parents and siblings of patients through the Multi-Generation Register to extract historical variables (i.e. before the current episode) on family members (see Appendix pp. 1–2 for details on all risk factors). From the National Crime Register, we obtained information on any previous violent conviction.

### Measurement of outcomes

Our primary outcome was the occurrence of suicide within one year. Suicides included undetermined deaths (i.e. ICD codes X60–84 and Y10–Y34) as is standard in the field of suicide research^22^. As the cause of death register covers more than 99% of deaths in Swedish residents, including those occurring outside Sweden, the loss of information on suicide was minimal^23^.

### Statistical methods

Statistical analysis was based on multivariable logistic regression (see below). The effects of non-suicide death and emigration within the follow-up period were ignored as the aim was to predict suicide within one year irrespective of whether these events occurred, and based only on information available at the time of episode. STATA (version 12) and R version 3.2.1 were used for all analyses. We reported our study based on the TRIPOD reporting guidelines for prediction models^24^. We considered allowing for competing risks but thought that the benefits would be outweighed by the additional complexity of the model given the number of censored individuals in the short follow-up is relatively small.

#### Risk factors

Based on existing evidence into socio-demographic, familial, and clinical factors^25^, we grouped variables a priori on the anticipated strength of association with the outcome in decreasing levels of priority^26,27^. All variables were categorised in this way in a protocol before any statistical analysis was carried out (see Appendix p.2 for description). Table 1 specifies variable groups. Length of first inpatient stay and number of previous episodes were dichotomised in a pre-specified way for ease of interpretation. Diagnostic information about individuals and parental factors was dichotomised to align how it is reported in clinical practice. Treatment information was categorised (previous 6 months). Past self-harm was also dichotomised, although it is possible that information on number of episodes might have been informative. However, we were concerned that this may not be available routinely (due to non-hospital episodes), and subject to measurement bias. Measures of income and deprivation were transformed into deciles to enable use in populations with differences in distribution of income and deprivation, and where different measures are commonly used. We did not test interactions due to limited statistical power between the 24 tested variables with lack of strong theoretical basis to examine specific ones.

**Table 1:** Baseline characteristics of the derivation sample (*n* = 58,771) diagnosed with schizophrenia-spectrum and bipolar disorders with grouping of suicide risk factors

Group	Variable	
1	Sex (male)	29,077 (49%)
1	Age at discharge (SD)	44 (13)
1	Previous violent crime	9212 (16%)
1	Previous drug use	7123 (12%)
1	Previous alcohol use	8897 (15%)
1	Previous self-harm	11,510 (20%)
1	Educational level	
	Primary	17,814 (35%)
	Secondary	26,449 (52%)
	Tertiary	6489 (13%)
1	Parental drug or alcohol use	5214 (11%)
1	Parental suicide	1417 (3%)
2	Diagnosis	
	Schizophrenia-spectrum disorders	36,755 (63%)
	Bipolar disorder	22,016 (37%)
2	Recent medication trethose with a schizophreniaatment (within preceding 6 months)	
	Mood stabiliser	10,390 (32%)
	Antipsychotic	18,401 (54%)
	Antidepressant	13,255 (39%)
	Drug and alcohol dependence	1030 (3%)
2	Inpatient at time of assessment	18,160 (31%)
2	Length of first inpatient stay > 7 days	24,532 (42%)
2	Number of episodes > 7	16,686 (28%)
3	Benefit receipt	37,210 (64%)
3	Deprivation (illustrative)	
	1st decile (lowest)	2793 (5%)
	5th decile	4862 (9%)
	10th decile (highest)	10,769 (19%)
3	Marital status: never married	34,506 (60%)
3	Personal income (illustrative)	
	1st decile (lowest)	5444 (9%)
	5th decile	9169 (16%)
	10th decile (highest)	2009 (3%)
3	Children in household	11,079 (19%)
3	Parental psychiatric hospitalisation	13,225 (28%)
3	Parental violent crime	3203 (7%)
3	Sibling violent crime	4028 (7%)
3	Comorbid depression	11,934 (32% of those with a schizophrenia-spectrum diagnosis)
3	Recent death of family member (within preceding 6 months)	953 (2%)

*Note*: Group 1 refers to variables included in model on the basis of previous evidence, Group 2 variables with strong evidence but needed validation, Group 3 variables with weaker evidence that we tested. Number (%) or mean (SD). Missing values are excluded from the denominator in the calculation of the percentages in the above table. Variables with substantial percentages of missing data: educational level (14%), parental drug or alcohol use (18%), parental violent crime (18%), benefit receipt (1.5%), deprivation (3.7%), marital status (2.2%), personal income (1.5%), parental psychiatric hospitalisation (18%), parental suicide (18%), recent treatment (54%) and recent death of family member (18%)

#### Missing data

Variables with more than 30% missing data were excluded (body mass index, other physical health variables, and IQ were not considered for this reason). An exception was made for the recent treatment information, which was unavailable before 2006 as the electronic register for prescription data was not in operation: the missingness mechanism was therefore known and thought to be unrelated to the missing values themselves. Missing data was imputed via multiple imputation with 20 imputations using a regression model that used as explanatory variables all other risk factors that were candidates for inclusion in the model, and the outcome variable^28^. Estimates of coefficients in the final prediction rule were obtained by pooling across imputations, using standard methodology^29^.

### Validation and goodness of fit

The patient cohort was split into separate model development and external validation datasets, to allow external validation using individuals not used in model development. The sample was split based on a stratified random selection of geographical regions^30^, based on the residential geographical location of the individual at the time of diagnosis with the external validation dataset comprising around one-fifth of the total sample. The number of regions selected for the validation sample was chosen to be large enough for a useful assessment of external validity to be conducted^31^ (see Appendix Table 1).

#### Statistical methods for validation

The internal validity of the model was assessed using bootstrapping to assess its predictive accuracy^32^. Bootstrapping was used to create 100 samples drawn with replacement from the derivation data-set; more bootstrap samples were not required as model performance measures were found to be very similar in different samples. A heuristic shrinkage estimate (model *χ*^2^–degrees of freedom]/model *χ*^2^) was calculated to assess the generalisability of the model^33^. Model performance was also assessed using the external validation sample. Predictive accuracy was summarised using the following measures: (1) the concordance index^34^ to assess discrimination (ability of the model to distinguish between those who do and do not die from suicide, with a value of one meaning perfect discrimination); (2) the Brier score^35^ for calibration (model goodness of fit—whether the predicted risk systematically off target, with zero meaning perfect calibration); (3) the net reclassification index^36^ (how well a model rightly or wrongly reclassifies patients compared with alternative models), and (4) sensitivity and specificity based on a 1% threshold of predicted probability. The choice of this 1% threshold was based on previous research that found 0.6% of schizophrenia-spectrum patients dying from suicide within one year of diagnosis^37^. These measures were calculated using the predicted probabilities obtained by averaging the predictions from each of the multiply imputed datasets, each applied to the final model. The proportions of predicted and observed events at different levels of predicted probability were compared using a calibration plot. A *p*-value of <0.1 was used to determine statistical significance for variable selection. On the suggestion of a peer reviewer, we also considered using lasso to address possible overfitting.

### Web calculator

We applied variable coefficients (Table 2) to develop a web calculator called OxMIS (Oxford Mental Illness and Suicide tool).

**Table 2:** Associations between prespecified risk factors and suicide in the derivation sample from the multiple regression model (after multiple imputation)

Variable	Odds ratio [95% CI]	*p*-Value
Sex—male	1.92 [1.58, 2.33]	<0.001
Age (per 10 years)	0.92 [0.85, 0.99]	0.02
Previous violent crime	0.78 [0.60, 1.02]	0.07
Previous drug use	1.09 [0.84, 1.41]	0.54
Previous alcohol use	1.29 [1.02, 1.63]	0.03
Previous self-harm	2.55 [2.09, 3.11]	<0.001
Educational level		
Upper secondary	1.24 [1.00, 1.53]	0.05
Post-secondary	1.68 [1.24, 2.28]	<0.001
Parental drug or alcohol use	0.70 [0.50, 0.99]	0.04
Parental suicide	1.75 [1.14, 2.69]	0.01
Recent treatment—antipsychotic	1.29 [0.98, 1.69]	0.07
Recent treatment—antidepressant	1.75 [1.29, 2.38]	<0.001
Inpatient at the time of assessment	2.95 [2.45, 3.55]	<0.001
Length of first inpatient stay > 7 days	1.23 [1.00, 1.50]	0.05
Number of previous episodes > 7	0.77 [0.61, 0.97]	0.03
Benefit receipt	0.83 [0.67, 1.02]	0.07
Parental psychiatric hospitalisation	1.20 [0.97, 1.48]	0.10
Comorbid depression	1.27 [1.03, 1.56]	0.03

### Ethics approval

Anonymized data was received from Statistics Sweden following Regional Research Ethics Committee approval at Karolinska Institutet (2013/5:8). The linkage code was destroyed when linkage was made. Thus no individual consent was required.

## Results

Of the cohort of 75,158 patients with schizophrenia-spectrum or bipolar disorder, 16,387 were assigned to the validation sample and the remainder to the derivation sample. Baseline characteristics of the derivation sample are shown in Table 1. In the derivation sample, 494 individuals (0.8%) died of suicide within 12 months of their patient episode. The validation sample was based on 16,387 patients, and 139 suicides.

### Predictors of suicide

The strongest predictors of suicide within 12 months included being an inpatient at the time of assessment/diagnosis (OR = 2.95, 95% CI 2.45–3.55), previous self-harm (OR = 2.55, 95% CI 2.09–3.11) and being male (OR = 1.92, 95% CI 1.58–2.33) (Table 2). The decline in probability of suicide was approximately linearly related to increasing age. The average estimate of the shrinkage heuristic from the bootstrapped samples was 96% so the coefficients from the development model were used without shrinkage (Table 2).

**Fig. 1:**
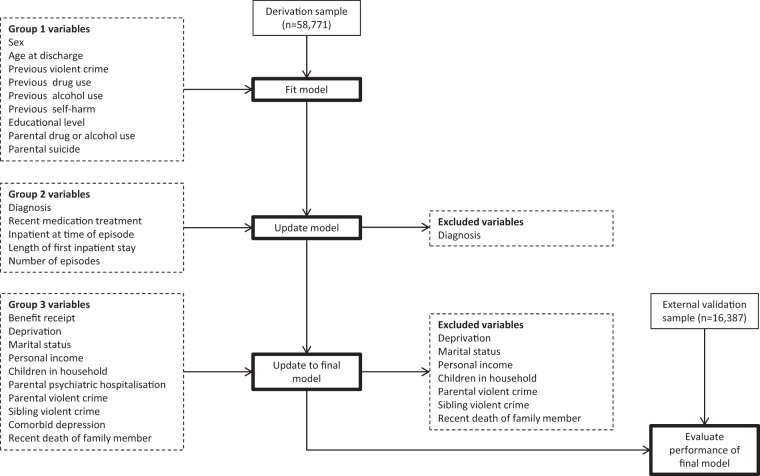
Flow chart of analytic approach to model development and validation

The final model was arrived at by including all the factor 1 variables, and those factor 2 and 3 ones that retained statistical significance in the multivariable analyses (see Fig. 1 for a flow chart of model development): sex, age, previous violent crime, drug use, alcohol use, self-harm, educational level, parental drug or alcohol use, parental suicide, recent antipsychotic treatment, antidepressant treatment, inpatient diagnosis, length of first inpatient stay, number of previous episodes, benefit recipiency, parental psychiatric hospitalisation, and comorbid depression (in those with schizophrenia-spectrum disorders). Variables included in the final 17-item model are shown in Table 2 (for full untransformed model coefficients, see Appendix Table 2).

**Fig. 2:**
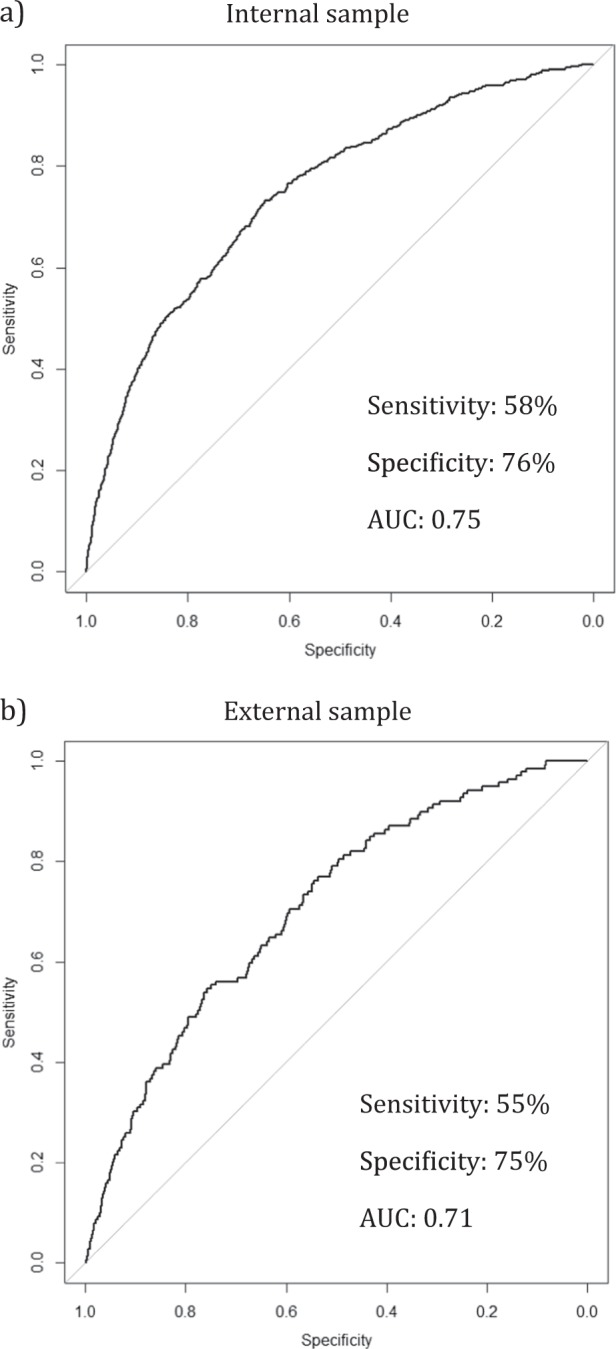
Suicide prediction model discrimination shown by receiver-operating characteristics curves. **a** Internal sample and **b** external sample. Sensitivity and specificity based on pre-specified 1% cut-off

The model showed good overall discrimination, based on both the results from internal validation using bootstrapping (c-index = 0.75 [95% CI 0.73–0.77], Brier score = 0.0082, net reclassification index = 0.67) and the results from external validation (c-index = 0.71 [95% CI 0.66–0.75], Brier score = 0.0084, net reclassification index = 0.51) (see Fig. 2 for ROC curves). When using the pre-specified 1% risk cut-off for suicide within one year, the sensitivity and specificity were 58% (95% CI 54–63%) and 76% (95% CI 76–76%), respectively, in internal validation. The sensitivity in external validation was slightly lower (55%, 95% CI 47–63%), with similar specificity (75%, 95% CI 74–75%). The positive predictive value and negative predictive value were 2% and 99%, respectively (see Table 3 for 2 × 2 tables).

**Table 3:** True and false positive and negatives from external validation (2 × 2 table)

	Suicide	No suicide	Total
High risk	77	4105	4182
Low risk	62	12,143	12,205
Total	139	16,248	16,387

**Fig. 3:**
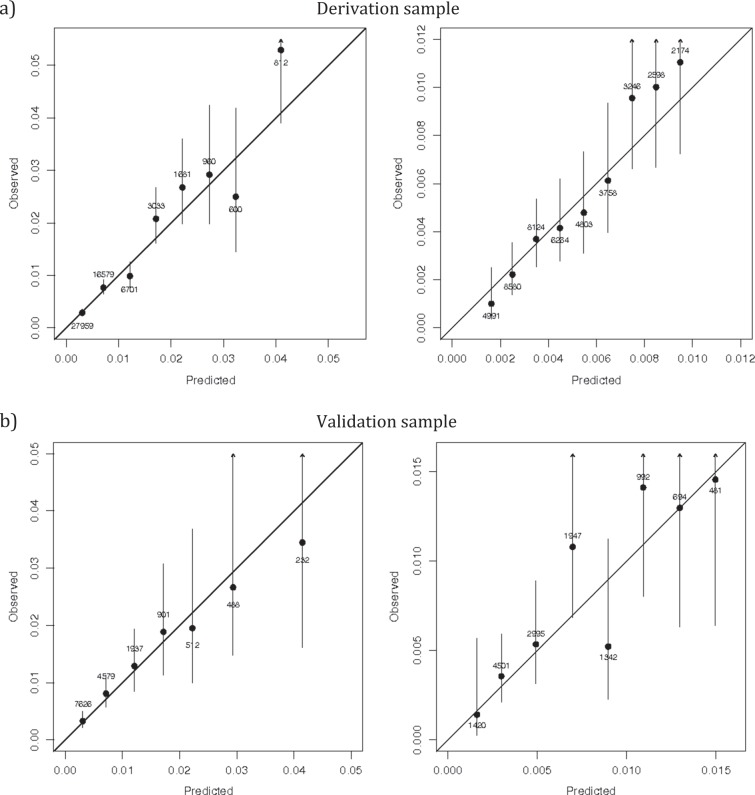
Calibration plots comparing predicted and observed risks of suicide in the derivation and validation sample of individuals with severe mental illness. **a** Derivation sample and **b** validation sample. Individuals are grouped by predicted probability, and points are labelled with the number of individuals in each grouping, and a 95% confidence interval for the proportion of events in each group

Calibration plots (Fig. 3) indicate adequate calibration of the predicted probabilities against observed proportions of suicide, based on estimated and observed risks being approximately similar, except for groups with few patients.

Lasso did not lead to any substantial improvement in fit, with levels of shrinkage similar to those indicated by the shrinkage heuristic from our original model (with a median reduction of 4%, the same as the shrinkage heuristic in our original model).

### Web calculator

A beta version of the online risk calculator for suicide (based on the coefficients in Appendix Table 2) can be found at http://oxrisk.com/oxmis for use by clinicians. If missing values are present, this calculator reports the upper and lower range of estimates of risk allowing for missing variables. The calculator presents probabilities scores, but not cut-offs (as discussed below).

## Discussion

This study presents the development and validation of a clinical risk score for suicide in individuals with severe mental illness. The validation sample was based on 16,387 patients, and 139 suicides. The score is based on 17 items, which are mostly routinely collected, including markers of illness severity and comorbidity, and recent medication. To our knowledge, this is the first such prediction score developed in individuals with schizophrenia-spectrum disorders and bipolar disorder. In the external validation, an overall c-index of 0.71 was reported using a cut-off, and it performed moderately well on calibration (with probability scores).

On the basis of the low positive predictive value and a sensitivity of 55% based on a pre-specified cut-off score of 1% suicide risk, we have removed the possibility of using it with a dichotomous risk category, and instead have made the tool available with the continuous probability score, and recommend that its use primarily complements individual needs-based suicide risk assessments. The use of a continuous probability score is supported by its calibration performance in external validation. The possibility of doing this is a clear advantage of our online risk calculator. This approach would allow for the floor of expertise to be raised, and provide a validated marker of suicide risk to be added into other health and psychosocial needs identified clinically. Further, it would allow for a discussion between clinicians and their patients and carers about their own risk level in a transparent manner. Even with a focus on probabilities, it is possible that someone with a predicted risk of 0% will die from suicide, and clinical judgement will be required to determine clinical management, and clear clinical indicators of risk not captured in the tool should take priority. All included risk factors were from routinely collected register data and are likely to be known for most patients without additional interviewing, thus the tool will have negligible additional resource implications. Additionally, certain variables have a broad categorical definition (education). Nevertheless, some items can be marked as unknown, and OxMIS provides a risk range, based on the lower and upper bound of possible responses.

An important consideration will be how clinicians will use such probability scores, and whether they will make any impact to clinical management. These are empirical questions that require further examination. However, one promising approach suggested by an expert review that is that low scores can assist clinical decision-making to preserve resources by excluding low risk individuals in an evidence-based way^8^. Although the negative predictive value based at the 1% cut-off is not very different from the pre-test probability, a tool provides a more transparent approach, and one which is flexible (i.e. clinical services can decide on different thresholds depending on local factors). Those identified using this suicide risk calculator with higher risk scores, assessment without clear links to improving clinical management will not reduce adverse outcomes. At the same time, it is necessary to have a validated risk assessment before consideration can be given to linking the score to an intervention^38^. Developing and validating a prediction model will necessarily precede the next step of what is done to manage it. Possible interventions that could be considered include increased surveillance, psychological interventions, and service-related changes. Non-harmful interventions should be prioritised.

One of the main reasons for not using cut-off scores is the low positive predictive value of 2%, which will mean that most individuals who will be labelled high risk do not subsequently die from suicide in the next year. Low positive predictive values have also been shown in risk assessment instruments in other populations^12^. In some settings, high risk persons may be subject to additional resources that may not be beneficial or necessary (such as inpatient admission). At the same time, using a cut-off of 1%, the tool accurately ruled out a large number of individuals—12,143 individuals in the validation sample of 16,387 or 74%—and hence could assist in preserving healthcare resources by limiting the number of more comprehensive suicide risk assessments. One of the challenges faced by screening tools— the prevention paradox that most suicides occur in low-risk individuals—did not clearly apply even if the categorical score was used. Of the suicides in the validation sample, 55% (77/139) were correctly identified as high risk but nevertheless 45% (62/139 suicides) were in the low-risk group. But another way of looking at this is that individuals with predicted suicide risks of <5% contributed to nearly half the suicides. This is one of the reasons why tools with categories (such as low/high) have been criticised^6^, and part of the rationale to remove categories in our online calculator. At the same time, statistics such as positive and negative predictive values depend on the threshold used, which we pre-specified in a protocol, but could be improved with different thresholds. Having a lower threshold (such as 0.5%) will lead to a much higher positive predictive value but at the cost of lower performance in other metrics. This underscores our decision to remove these categories in OxMIS. In summary, designing any prognostic tool and deciding on how it will be used will require an inevitable balance between overdiagnosis (false positive predictions) and missing a diagnosis (false negative predictions). Both types of error can cause significant harm. Moreover, resources for supporting prevention of suicide are limited, and so targeting of resources to high-risk patients is important. Our tool is designed to be used in conjunction with assessing other health and psychosocial needs, which would allow additional detection of high-risk individuals and in discussion between clinicians, patients and carers.

In developing the score, risk factors for suicide were tested on a sample of 58,771 patients using a multivariable logistic regression model. This provides more precision than in previous studies to markers of suicide in patients with severe mental illness^25,39^ and contributes to the growing evidence on risk factors for suicide^16^. The strongest risk factors were being male (OR = 1.92, 1.58–2.33], being discharged from inpatient care (OR = 2.95, 2.45–3.55), previous self-harm (OR = 2.55, 2.09–3.11) and parental suicide (OR = 1.75, 1.29–2.38). The inpatient risk factor adds to the evidence that points towards the importance of improving the monitoring and management of patients in the early period post-hospital discharge with prompt follow-up, access to specialist substance misuse services, and addressing the physical safety of the local environment^40,41^. Social factors, such as highest educational level, were associated with future suicide risk^42^. Other risk factors identified in our cohort, such as recent treatment with antipsychotics and antidepressants, are likely to be markers of illness severity, and randomised controlled trials, which are not subject to confounding by indication, have not shown increased suicide risk in both medication classes^43,44^. The positive association with previous alcohol use and comorbid depression further highlights the need to treat co-occurring conditions^39^. The findings of elevated risk in individuals who have previously self-harmed are consistent with population studies in Denmark^2^ and Sweden^45^. We investigated whether the diagnosis (schizophrenia-spectrum vs. bipolar) was a candidate for inclusion in the model but there was no significant difference in risk by diagnosis in multivariable models. Other models were considered, such as random forest and stochastic gradient boosting, but do not provide a specific prediction equation that can be used for out-of-sample prediction, and hence cannot be translated into an online calculator. Presenting the effects of individual risk factors increases face validity as users are able to see the contribution of factors (especially modifiable ones) to risk prediction. We tested using a lasso to address possible overfitting but found no material improvement in fit.

A review of risk assessment tools found that almost all examine self-harm rather than suicide as an outcome, base their variable selection on face and content validity rather than empirical derivation, and are used in individuals who have self-harmed^8^. Three empirically derived tools, two Manchester self-harm rules and the RESH score, have been developed to assess risk of repeat self-harm in individuals presenting with self-harm to emergency departments. Other tools have been developed with specific populations, such as army veterans, and used a wide range of 421 predictors in model derivation^46^. A recent study aimed to use electronic health records to predict suicidal behaviour in any patient accessing healthcare in two Boston hospitals using more than 100 diagnoses and 100 medications in their models^47^. This US study used a random split to generate a validation sample, which meant that both samples would have been statistically equivalent and the validation likely to perform similar to the discovery sample. Further, it created a model that uses laboratory findings, and is therefore not scalable to community and other settings. In contrast, our score uses mostly routinely collected factors in mental health services, and we used an external validation that was geographical split, rather than randomly done. Finally, a tool with good performance measures has been designed to predict suicidal ideation and attempts^48^, and tested in adolescents, individuals in an antidepressant trial, and attending emergency departments, but not in specific diagnostic groups like schizophrenia-spectrum and bipolar disorders, and not for completed suicide. Some limitations of the current model should be noted. A further limitation is that we did not test information about symptoms, including suicidal thoughts and plans, which were not available in the registers used. Whether these items improve prediction incrementally when the other risk factors are already in a model is an empirical question, and may come with additional costs by making a tool longer and potentially requiring additional assessment. Further, the fluctuating nature and high prevalence of certain symptoms (including suicidal ideas) may mean that their predictive value is low. At the same time, it is possible that additional factors could improve this score, which future research can consider^42^. Prediction based on a narrow set of 4–5 risk factors has been shown to have limited utility in general population settings^49^, but the OxMIS score includes more items (17 in total) than current approaches and has been derived in a specific clinical population with higher risks than community persons. Missing data for some risk factors required multiple imputation to be used for model development. Although this allowed a larger sample to be used in model fitting, we cannot rule out that the model performance may have been better if complete data had been available. Finally, this model has few dynamic variables (recent medication use, past drug use disorder, past alcohol use disorder, comorbid depression, and possibly previous self-harm, and inpatient status) and this is a limitation, and new technologies may assist in developing new scores that reliably incorporate more dynamic risk factors. However, more complex models can risk over-fitting predictions to the specific dataset so that prediction accuracies are greatly reduced when applied to new populations, and may reduce the ability to facilitate discussions between clinicians and their patients about their risk in a transparent manner.

Before implementation of any such tool in clinical practice, external validation should be undertaken, and also examining how interventions can be linked to this risk calculator. Such external validation will provide feedback on its acceptability in clinical practice and whether probability scores supplement clinical decision-making, although data will not be harvested from the online tool as it is not known who will use it. Researchers may use OxMIS to provide baselines risks for clinical research studies. Ultimately, though, testing in RCTs will be required to see whether its use will reduce suicide rates. Furthermore, a prediction score for risk of non-fatal self-harm could be developed in this patient population.

In summary, we have developed and validated a clinical risk prediction score in individuals with schizophrenia-spectrum or bipolar disorder with good measures of discrimination and calibration. This has been translated into an online calculator providing probability scores, which can be used by healthcare staff to assist in clinical decision-making.

## Supplementary information

supplementary Information
