# Supplementary material for: The prediction of suicide in severe mental illness: development and validation of a clinical prediction rule (OxMIS)
Source: Transl Psychiatry. 2019 Feb 25;9:98. doi: 10.1038/s41398-019-0428-3 (PMC6389890; doi:10.1038/s41398-019-0428-3)
Supplement: Supplementary file 1 — supplementary Information [file 41398_2019_428_MOESM1_ESM.pdf]

Clinical prediction rules for persons diagnosed with an episode of severe mental illness for risk of violent crime and suicide – statistical analysis plan

Study Summary

Design: Retrospective cohort study.

Data: Swedish databases linking hospital, crime, prescriptions, family relationships, housing and socio-economic status/deprivation data.

Participants: The study cohort is defined as individuals aged 15-65 with a diagnosis of any major psychosis (including bipolar disorder, schizophrenia, schizophrenic spectrum, other psychotic illness), obtained from hospital records that encompass both inpatient and outpatient visits, during the study period.

Study period: Psychiatric episodes ('diagnoses') that occurred between 1st January 2001 and 31st December 2008 will be used to develop the model: one episode per person, chosen at random. Data on most risk factors, including prior convictions and family history, are available for the lifespan of the individual, and are therefore not subject to the same 2001 start date for diagnosis. An exception is treatment history, which is available from 2005 onwards.

Risk factors: Include demographic characteristics (including gender, age, educational level, birth country), previous offending, drug and alcohol addiction, familial factors (parental and sibling illness, criminality and suicide), treatment history and a measure of deprivation (see Appendix for complete list).

Primary outcomes: Two outcomes are specified: (i) any violent crime within 12 months of diagnosis; (ii) suicide within 12 months of diagnosis.

Outputs: The main objective is to produce models that estimate the probability of events for the two primary outcomes, with appropriate measures of predictive accuracy.

These models will be used to obtain scoring systems for the calculation of risk to be used prospectively at time of diagnosis. As part of the study, we will investigate whether it is appropriate to assign the resulting probabilities to discrete categories, such as low/medium/high risk.

Statistical Analysis

Statistical analysis will be based on logistic regression, adjusting for risk factors, in each of two scenarios:

(i) Violent crime within 12 months, from time of diagnosis

(ii) Suicide within 12 months, from time of diagnosis.

Here, 'diagnosis' refers to episodes of psychosis for which a diagnosis was recorded within the period 1st January 2001 to 31st December 2008. The terms 'diagnosis' and 'episode' are used interchangably in this document.

The method of analysis will be the same in both scenarios, so for clarity the explanation below refers to scenario (i).

In each case, the follow-up period represented in the data-set lasts for at least 12 months after diagnosis, and so it is not necessary to consider the possibility of censoring when performing the analysis.

Random selection of diagnoses

Given a starting data-set of all patients and all patient episodes (diagnoses) between 1/1/01 and 31/12/08, patient episodes will be selected at random as follows:

- Select at random, with equal probability, one inpatient or outpatient visit per patient.
- Note that some patients have more than one inpatient or outpatient visit on the same day, and some inpatient or outpatient visits comprise more than one diagnosis; only one inpatient or outpatient visit per day will be considered for random selection (if an inpatient visit and an outpatient visit are listed contemporaneously, the inpatient visit will be used).
- Extraneous episodes that are recorded as occurring within the period of an inpatient stay (because of recording errors or otherwise) will be identified in the data-set as having a date of episode that is earlier than lower-numbered episodes for the same individual; these will also be removed before the random selection step is made.
- If the inpatient or outpatient visit comprises a schizophrenia-spectrum diagnosis (with or without a bipolar diagnosis), this is considered as the diagnosis at time of selection; otherwise bipolar is the diagnosis at time of selection.
- For inpatient stays, the assumed date of diagnosis is the date the inpatient stay ends.
- The randomly selection of diagnoses defines the final data-set for analysis, i.e. there will be no subsequent resampling of diagnoses.

Adjustment for risk factors

Risk factors will be considered in three groups, of decreasing levels of priority. The 'List of risk factor variables' table in the Appendix specifies the group to which each variable is assigned.

Group 1 consists of variables that will be included in the statistical model regardless of statistical significance. These include demographic characteristics that it is necessary to include to ensure the model has face validity, and other risk factors strongly suspected on the basis of previous research to be associated with the two outcome measures.

Group 2 consists of variables likely to show an association with outcomes but which are not required to be included to achieve face validity. This group includes measures of prescribed treatment, a categorical variable indicating the diagnosis of the individual and proxy measures of disease severity, such as whether the diagnosis occurred while the patient was an inpatient or an outpatient. The model will use a backwards stepwise selection procedure to determine whether to retain these variables in the model, with Group 1 variables always retained and Group 2 variables sequentially rejected in order of p-value until no group 2 variables remain that have p-values greater than 0.1 (Royston and Sauerbrei, 2008).

Group 3 consists of variables for which there is weaker prior evidence to suggest that they will be associated with outcomes. This group includes a deprivation measure, a measure of marital status, an income measure and several variables relating to parental outcomes or characteristics. The model will use a backwards stepwise procedure to determine whether to retain these variables, holding fixed any variables from Groups 1 and 2 that are already included. Group 3 variables will be retained only if they have p-values less than 0.1.

This strategy of risk factor adjustment recognises that the final model must demonstrate face validity, whilst simultaneously allowing the inclusion of additional risk factors if they show an association with outcome variables. The variables are considered in three groups in this way to recognise that a parsimonious model is preferable (i.e. easier to use in practice), provided that it has acceptable predictive ability.

The same set of candidate covariates will be used for both models (barring the few exceptions listed in Appendix 1).

Clustering by family

It is likely that the data-set will exhibit clustering by family: sibling or parent-child relationships for which a diagnosis of major psychosis occurs for more than one member of

the same family. If this is the case, the model will include a single random effect term to take into account family membership.

Competing risks

A competing risk model, which might model jointly the probability of two or more events (such as violent crime and death), will not be used for the main analysis for two main reasons. Firstly, the outcome variable will be analysed as binary, and secondly, every individual in the data-set is followed up for at least twelve months after the initial time point. The main concern in failing to allow for competing risks (in this case, death) is inappropriately treating individuals as censored if they experience the competing risk event (Putter, 2007). However, in this study the length of follow means that there are no individuals lost to follow up during the twelve-month period of interest, and so any individual who has experienced the competing risk before the outcome can simply be assigned a zero value for the outcome.

Linearity and additivity assumptions

Smoothed partial residual plots will be used to check the assumption that continuous covariates are linearly associated with the log odds of the outcome. If there is a clear departure from normality, fractional polynomial terms will be included, as appropriate (Royston and Sauerbrei, 2008). Covariates that may have an extremely skewed distribution, such as the number of previous hospital admissions, may be dichotomised if model checking reveals poor fit when the variable is included as a continuous measure; it is not possible to anticipate the extent to which this will be necessary, so this will be a pragmatic decision based on the goodness-of-fit of the model, with the intention of guarding against extreme values of the covariate that may unduly affect patterns of associations or measures of predictive accuracy. Interactions between covariates will not be considered as possible predictors.

Missing data

Covariates that have more than 30% missing data will be excluded (an exception is made for the recent treatment variables, for which there is a particular pattern of missing data – see subsection below). Missing data on covariates with at most 30% missing data will be imputed via multiple imputation (with twenty imputations) using a regression model that uses other risk factors and the outcome variables as explanatory variables (Sterne et al., 2009). Estimates of coefficients in the final prediction rule will be combined across imputations, using standard methodology (Barnard and Rubin, 1999). It is possible that the

variable selection procedure would give different sets of variables in different imputations of the data-set; for this reason, the 'RR' method described by Wood et al. (2008) will be used. In this method, summary estimates are computed by combining information across all imputed data-sets at each stage as part of a single variable selection process. The missing indicator method will not be used even though treatment data may reasonably be regarded as missing completely at random during the period 2001-2004. In observational studies, this method has been shown empirically to bias the effect size estimates of other variables in the model even if the missing completely at random assumption holds (Groenwold et al., 2012).

Recent treatment variables

Recent treatment variables are expected to have a high proportion of missing data values because information on treatment within the preceding six months is not available for episodes dated before the beginning of 2006. For this reason, it appears reasonable to regard the missing values for these four variables as missing completely at random; in this scenario, there is evidence that multiple imputation models demonstrate good performance, and are preferable to complete-case analysis, even when the proportion of missing data is high (White and Carlin, 2010; Lee and Carlin, 2012). Therefore multiple imputation will be performed on these variables in the same way as described in the section 'Missing data'. As a sensitivity analysis, the final model (using the same set of covariate models) will be fitted using complete-case data from 2006-2008 only, and any substantial differences in point estimates noted. Standard errors of estimates in this complete-case analysis are expected to be larger owing to the smaller sample size.

Validation and goodness of fit

To test the external validation of the model, a sub-sample of geographical regions (the 'validation sample') (Bleeker et al., 2003), based on the residential geographical location of the individual at the time of diagnosis, will be selected and removed from the data-set used to fit the model. Geographical regions are shown in Appendix 2. Regions are primarily based on the counties of Sweden, derived from the first two digits of the SAMS code. Exceptions are the municipalities of Gothenburg and Malmö, which are separated from their respective counties, and Stockholm municipality, which is separated from its county and sub-divided into northern and southern parts by identifying each SAMS area with the historical province in which it is located. Regions are allocated to four groups, which are proxy measures of urban/rural status: the four urban areas (Group 1); the three counties in which the urban areas are located (Group 2); four counties with low population (Group 3); and all other counties (Group 4). The external validation sample will be selected by

randomly, with equal probability, choosing one region from each of the first three groups, and selecting sequentially from the fourth group until a minimum of 180 violent crime cases in total has been reached (see Appendix 2 for details). This guarantees that the number of cases in the external validation sample will be large enough for a useful assessment of external validity to be made (Vergouwe et al., 2005).

All remaining data (the 'model development sample') will be used in the development of the model, to determine which risk factors are to be included. As one of the objectives is to develop a model that can be used externally (in different geographical settings), the geographical location itself is not considered as a candidate predictor in the model.

Once a 'final' model has been found using the steps outlined above, its internal validation will be assessed using bootstrapping to assess its predictive accuracy (Harrell et al., 1996). The bootstrapping step will use repeated resampling of families (rather than individuals – see section 'Clustering by family') in order to preserve within-family correlation. The performance of the model will also be assessed using the external validation sample. Predictive accuracy will be summarised using several summary measures, including the concordance index (Harrell et al., 1982), the Brier score (Brier, 1950) and the net reclassification index (Pencina et al., 2008). The proportions of predicted and observed events at different levels of predicted probability will be compared using a calibration plot.

If it is deemed appropriate to split the predicted probabilities into just two categories ('Low' and 'High'; see below), sensitivity, specificity and positive and negative predictive values will also be reported.

Presentation of findings

The main output of the model will be a predictive probability, indicating the probability of occurrence of the outcome of interest within 12 months. The estimated coefficients of individual risk factors will be examined with a view to: (i) simplifying the prediction rule in order to make it easier to use in practice, for example by using integer-valued coefficients (including possibly resetting Group 1 variables with extremely poor predictive power to have a coefficient of zero) or even by dichotomising numerical variables, provided this does not compromise its predictive accuracy; and (ii) justifying a categorisation of the predicted probability into risk categories (for example 'Low risk' (<5%), 'Medium risk' (5-10%) and 'High risk' (>10%)). The latter would also benefit from an assessment of the number and characteristics of individuals that fall into the proposed categories in the model development sample and the validation sample.

Generalisability

Variables are defined in such a way as to help the generalisability of the model to other settings (see table below). For instance, deprivation will be included in the model in deciles rather than as a continuous variable in order that other deprivation measures that are used in other settings can be included in the same model to obtain predicted probabilities. When the final model is used for prediction, it is possible that some variables that are included as covariates may be missing. We will provide guidelines for using the model for prediction in this scenario, for example by using mean imputation of the missing variable.

References

Barnard J, Rubin DB. Small-sample degrees of freedom with multiple imputation. Biometrika 1999; 86: 948-955.

Bleeker SE, Moll HA, Steyerberg EW, Donders ART, Derksen-Lubsen G, Grobbee DE, Moons KGM. External validation is necessary in prediction research: a clinical example. Journal of Clinical Epidemiology 2003; 56: 826-832.

Brier GW. Verification of forecasts expressed in terms of probability. Monthly Weather Review 1950; 78: 1-3.

Groenwold RHH, White IR, Donders ART, Carpenter JR, Altman DG, Moons KGM. Missing covariate data in clinical research: when and when not to use the missing-indicator method for analysis. Canadian Medical Association Journal 2012; 184: 1265-1269.

Harrell FE, Califf RM, Pryor DB, Lee KL, Rosati RA. Evaluating the yield of medical tests. Journal of the American Medical Association 1982; 247: 2543-2546.

Harrell FE, Lee KL, Mark DB. Multivariable prognostic models: Issues in developing models, evaluating assumptions and adequacy, and measuring and reducing errors. Statistics in Medicine 1996; 15: 361-387.

Lee KJ, Carlin JB. Recovery of information from multiple imputation: a simulation study. Emerging Themes in Epidemiology 2012; 9: 3.

Pencina MJ, D'Agostino Sr RB, D'Agostino Jr RB, Vasan RS. Evaluating the added predictive ability of a new marker: from area under the ROC curve to reclassification and beyond. Statistics in Medicine 2008; 27: 157-172.

Putter H, Fiocco M, Geskus RB. Tutorial in biostatistics: competing risks and multi-state models. Statistics in Medicine 2007; 26: 2389-2430.

Royston P, Sauerbrei W. Multivariable model-building. 2008. Chichester: Wiley.

Sterne JAC, White IR, Carlin JB, Spratt M, Royston P, Kenward MG, Wood AM, Carpenter JR. Multiple imputation for missing data in epidemiological and clinical research: potential and pitfalls. BMJ 2009; 338: 157-160.

Vergouwe Y, Steyerberg EW, Eijkemans MJC, Habbema DF. Substantial effective sample sizes were required for external validation studies of predictive logistic regression models. Journal of Clinical Epidemiology 2005; 58: 475-483.

White IR, Carlin JB. Bias and efficiency of multiple imputation compared with complete-case analysis for missing covariate values. Statistics in Medicine 2010; 29: 2920-2931.

Wood AM, White IR, Royston P. How should variable selection be performed with multiply imputed data? Statistics in Medicine 2008; 30: 3227-3246.

Appendix 1: List of risk factor variables

Time varying covariates (e.g. age) are used based on the value at diagnosis. Variables referring to 'previous' events are based on events that happen on or before the date of diagnosis. 'Group' (see section 'Adjustment for risk factors' in main document) refers to the model for violent crime; group allocation for the suicide outcome is given in brackets, where this is different. A dash (-) indicates a variable that will not be included in the model.

Variable	Group	Type	Notes
Sex	1	Binary	Male / female
Age	1	Continuous	
Previous violent crime	1	Binary	Any violent crime conviction before date of diagnosis.
Previous drug use	1	Binary	Use disorder or dependence
Previous alcohol use	1	Binary	Use disorder or dependence
Previous self-harm	1	Binary	
Educational level	1	Categorical	Seven categories, ranging from less than 9 years primary and lower secondary education (level 1) to postgraduate education (level 7); converted to three categories for analysis (primary/lower secondary education (1/2), upper secondary education (3/4), post-secondary education (5/6/7))
'Immigrant descent'	1	Binary	Either individual born abroad, or either parent born abroad
Parental drug or alcohol use	1	Binary	Parental previous drug use and parental previous alcohol use will be combined into a single variable
Parental violent crime	1 (3)	Binary	
Sibling violent crime	1 (3)	Binary	
Diagnosis	2	Binary	Classified as either 'bipolar' or 'schizophrenia' (the latter also covers the schizophrenic spectrum)
Recent treatment – drug class	2	Categorical	Four categories (each treated as binary: 'mood', 'antipsychotic', 'antidepressant', 'dependence'); defined as any prescription collected in the six months prior to diagnosis
In/outpatient diagnosis	2	Binary	Indicator variable of whether patient was an inpatient or an outpatient at time of diagnosis
Length of first inpatient stay	2	Discrete	Measured in days, included in model as a binary variable (up to 7 days versus 8 or more days); relevant for inpatients only
Number of episodes	2	Discrete	Includes both inpatient admissions and contacts with health services as

			an outpatient; included in model as a binary variable (up to 7 versus 8 or more)
Benefit receipt	3	Binary	Comprises both welfare and disability benefit
Deprivation	3	Continuous	A standardised continuous measure, calculated relative to the general population, and expressed in deciles and included in model as a discrete variable
Marital status	3	Categorical	Seven categories, converted to binary (unmarried versus all other categories combined)
Personal income	3	Categorical	Calculated relative to the general population, and expressed in deciles and included in model as a discrete variable
Children in household	3	Binary	Zero / non-zero
Parental psychiatric hospitalisation	3	Binary	Use 'care at psychiatric clinic' for this
Parental suicide	3 (1)	Binary	
Comorbid depression + schizophrenia	- (3)		Any prior diagnosis of depression; among patients with a schizophrenia diagnosis only
Recent death of family member (< 6 months before diagnosis)	3	Binary	Any parent or sibling

BMI, physical characteristics and IQ were considered as possible Group 2 variables, but subsequently excluded based on the high proportion of missing data

Appendix 2: Classification of regions for selection of external validation sample

Group 1	Group 2	Group 3	Group 4
Major urban centres	Counties with major urban centres removed	Counties with small population	Counties with medium population
1 Stockholm City North	1 Stockholm County Other	7 Kronoberg	3 Uppsala
1 Stockholm City South		9 Gotland	4 Södermanland
12 Malmö	12 Skåne Other	10 Blekinge	5 Östergötland
14 Gothenburg	14 Västra Götaland Other	23 Jämtland	6 Jönköping
			8 Kalmar
			13 Halland
			17 Värmland
			18 Örebro
			19 Västmanland
			20 Dalarna
			21 Gävleborg
			22 Västernorrland
			24 Västerbotten
			25 Norrbotten

Regions will be selected at random, with equal probability, as follows:

- One region from Group 1
- One region from Group 2 (under the constraint that no more than one region in Groups 1 and 2 from the same county can be selected)
- One region from Group 3
- Sequentially select from Group 4 until the number of violent crime events in the sample reaches at least 180
